# Secondary invasion un‐undefined: The importance of consistent and clear terminology for classifying unique invasion phenomena

**DOI:** 10.1002/ece3.3964

**Published:** 2018-04-27

**Authors:** Luke S. O'Loughlin, Peter T. Green

**Affiliations:** ^1^ Department of Ecology, Environment and Evolution La Trobe University Bundoora VIC Australia; ^2^ Fenner School of Environment and Society The Australian National University Canberra ACT Australia

**Keywords:** facilitation, invasion complex, invasion success, invasional meltdown, population release, true entry

## Abstract

**Linked Article**: https://doi.org/10.1002/ece3.3966

Invasion ecology boasts an impressive array of hypotheses, frameworks, and concepts that have been developed to explain how non‐native species establish in and dominate native ecosystems (“invasion success”) (Blackburn et al., 2011; Catford, Jansson, & Nilsson, 2009). A key reason why so many connected and complementary ideas have been proposed is the high degree of context‐specificity intrinsic to the patterns and processes that invasion ecology investigates. In a recent article in *Ecology and Evolution* (O'Loughlin & Green, 2017), we proposed (1) a novel framework for understanding where invasion success of one non‐native species is contingent on other invaders altering properties of the recipient community, and (2) argued that the term “secondary invasion” be used to describe that particular phenomenon. Using examples, we showed that this term has previously been used to describe disparate phenomena in invasion biology. In response, Pearson, Ortega, Runyon, and Butler (2018) argue that while our proposed framework makes a meaningful contribution to the field, our use of the term to describe a very narrow phenomenon is problematic and unfounded. Here, we reply to arguments made by Pearson et al. (2018) and reaffirm our position (O'Loughlin & Green, 2017) that “secondary invasion” be applied specifically in cases of invader‐facilitated invasions.

Their first point is that we have redefined a term in such a way as to make it less useful by making it too narrow. We contend (again) that “secondary invasion” has never been formally defined in invasion ecology, and we further point out that Pearson et al. (2018) have not cited any papers where a definition is given. While individual researchers may use the term consistently and with what they feel is clear meaning, it is obvious from the broader literature that “secondary invasion” continues to be used to describe no less than five unique invasion phenomenon (see Table 1 in O'Loughlin & Green, 2017). We appreciate that for researchers focused on the impacts of invasive plants and their management on native plant communities (e.g., Pearson, Ortega, Runyon, & Butler, 2016), “secondary invasion” is widely understood to mean where invasive species establish following management intervention of a different invader (what we referred to as “management‐mediated invasion”). However, this is quite different from how others have used the term or even described that same process outside of invasive plant management (e.g., Albaina et al., 2016; Baldwin, Carpenter, Rury, & Woodward, 2012; Flory & Bauer, 2014; Green et al., 2011; O'Loughlin & Green, 2016). We maintain that there is no intrinsic common feature that would justify the continued undefined use of “secondary invasion” to simultaneously label multiple and quite dissimilar invasion phenomena (O'Loughlin & Green, 2017).

Their second point is that we based our definition on precedence. We agree that precedence alone is not sufficient justification for defining nomenclature, which is why our decision to settle on a particular definition was based on other factors. We discounted some uses based on the existence of alternative terms that are already commonly used in the literature (e.g., “secondary spread”; see Richardson, Pysek, & Carlton, 2011) and critically evaluated the remaining uses for where the term seemed most applicable based on how “secondary invasion” is used outside of ecology (because within invasion ecology, there is no clear dominant). We presented the alignment of our definition with the first ever use of “secondary invasion” in the title of a broadly ecological article (Wicklow, Bennett, & Shotwell, 1987) as a point of interest, not of justification.

Third, Pearson et al. (2018) took us to task over our scholarship. As they point out, the terms “secondary invasion” or “secondary invader” have been used before (in fact, the oldest we have found is by Schmieder (1927)), but on careful reading, it is obvious the authors typically used the term as a one‐off in the discussion, and usually in reference to the appearance of one native species or another in a successional sequence of native species. On the contrary, we were obviously looking for papers dealing specifically with non‐native species, and where “secondary invasion” was a meaningful part of the focus, questions, or conclusions of the research. That was why we focused our search on the title, abstract, and keywords. We point out that ironically, Pearson et al.'s advocacy for a broad definition of secondary invasion, drawing on traditions from the decades‐old succession literature, is itself rooted in precedence.

Fourth, Pearson et al. (2018) argue that our review generated only two cases of what they describe as obligatory facilitation of secondary invaders and that rarity negates the broader appeal of some of our ideas. We hasten to point out that our paper was *not* a review and that we simply chose examples to illustrate our points (which included more than the two case studies we chose to detail). The question as to whether secondary invasion as we have defined it is common or rare is still open—to review.

Fifth, and somewhat confusingly, Pearson et al. (2018) have presented a key component of our framework, and a major distinction we make in our paper, as an independent argument against our own. They contend non‐native species are so rarely precluded entry that the bulk of invader‐facilitated invasions are really primary invaders (arriving first, concurrent, or later—again, a point we made) facilitating other non‐native species that are not completely excluded from the ecosystem. This is the *exact* distinction we make in our paper to the extent that there is an entire section dedicated to this difference (see “Demonstrated secondary invasions at different stages of the invasion pathway”, with accompanying Figures 4 and 5 in O'Loughlin & Green, 2017), and it forms our key conclusion that we should recognize two distinct models of secondary invasion (“true‐entry” and “population‐release”) (also see O'Loughlin & Green, 2015).

Finally, we disagree on terminology. Pearson et al. (2018) propose “invader‐contingent invasion” and “invader‐facilitated invasion” as more appropriate synonyms for what we describe as “true‐entry secondary invasion” and “population‐release secondary invasion,” respectively. We consider their terms to be far less useful than ours as they fail to distinguish “invasive species” from simply “non‐native species,” do not make clear reference to the stage of the invasion pathway facilitation occurs, and the terms themselves are not intuitively different. Within the context of invasion ecology, “contingent” and “facilitated” are both used to refer to processes that occur only in certain circumstances (i.e., they describe the same idea—invasion success is *dependent* on something). We maintain that our terms and definitions are far more appropriate because they capture accurately the dual aspects of secondary invasion (invader‐facilitation per se, and the stage at which the interactions are important) without confusing “invasion success.”

In summary, Pearson et al. (2018) argue that narrowly defining “secondary invasion” is unwarranted because of some supposed benefits to the ecology of a more generic and less‐restrictive use of the term. We reiterate a point made in our original paper that using vague, inconsistent, and unspecified language leads to uncertainty, miscommunication, and loss of information (Regan, Colyvan, & Burgman, 2002). When terms appear to be obvious and intuitive, they are quickly interpreted in different ways, leading to ambiguity, and eventually so much confusion and redefinition that the concept becomes meaningless (Cottee‐Jones & Whittaker, 2012). The vagueness and ambiguity around the term “secondary invasion” exist, in part, because it was never defined as a clear concept in the first instance. Our detailed framework clearly accounts for the diversity of biotic and abiotic factors that may influence invasion and recognizes specific types of secondary invasion. Importantly, our paper provides that first conceptual definition of an *undefined* term.

## CONFLICT OF INTEREST

None declared.
